# Hazards of Using *Masking Protocols* When Performing Ligand Binding Assays: Lessons From the Sigma-1 and Sigma-2 Receptors

**DOI:** 10.3389/fphar.2020.00309

**Published:** 2020-03-13

**Authors:** Haider Abbas, Preeti Borde, Gary B. Willars, David R. Ferry, Stephen T. Safrany

**Affiliations:** ^1^ School of Pharmacy, University of Wolverhampton, Wolverhampton, United Kingdom; ^2^ Oncology Department, New Cross Hospital, Wolverhampton, United Kingdom; ^3^ School of Medicine, RCSI-Bahrain, Adliya, Bahrain; ^4^ Department of Molecular and Cell Biology, University of Leicester, Leicester, United Kingdom; ^5^ Gastrointestinal Oncology Strategy, Eli Lilly, Indianapolis, IN, United States

**Keywords:** dextrallorphan, di-*O*-tolyl guanidine, equilibrium binding, masking, (+) pentazocine, sigma-1, sigma-2, TMEM97

## Abstract

Sigma-1 and sigma-2 receptors are emerging therapeutic targets. Although the molecular identity of the sigma-2 receptor has recently been determined, receptor quantitation has used, and continues to use, the sigma-1 selective agents (+) pentazocine or dextrallorphan to *mask* the sigma-1 receptor in radioligand binding assays. Here, we have assessed the suitability of currently established saturation and competition binding isotherm assays that are used to quantify parameters of the sigma-2 receptor. We show that whilst the sigma-1 receptor mask (+) pentazocine has low affinity for the sigma-2 receptor (K_i_ 406 nM), it can effectively compete at this site with [³H] di-*O*-tolyl guanidine (DTG) at the concentrations frequently used to mask the sigma-1 receptor (100 nM and 1 µM). This competition influences the apparent affinity of DTG and other ligands tested in this system. A more troublesome issue is that DTG can displace (+) pentazocine from the sigma-1 receptor, rendering it partly *unmasked*. Indeed, commonly used concentrations of (+) pentazocine, 100 nM and 1 µM, allowed 37 and 11% respectively of sigma-1 receptors to be bound by DTG (300 nM), which could result in an overestimation of sigma-2 receptor numbers in assays where sigma-1 receptors are also present. Similarly, modelled data for 1 µM dextrallorphan show that only 71–86% of sigma-1 receptors would be masked in the presence of 300 nM DTG. Therefore, the use of dextrallorphan as a masking agent would also lead to the overestimation of sigma-2 receptors in systems where sigma-1 receptors are present. These data highlight the dangers of using masking agents in radioligand binding studies and we strongly recommend that currently used masking protocols are not used in the study of sigma-2 receptors. In order to overcome these problems, we recommend the use of a cell line apparently devoid of sigma-1 receptors [e.g., MCF7 (ATCC HTB-22)] in the absence of any masking agent when determining the affinity of agents for the sigma-2 receptor. In addition, assessing the relative levels of sigma-1 and sigma-2 receptors can be achieved using [³H] DTG saturation binding followed by two-site analysis of (+) pentazocine competition binding with [³H] DTG.

## Introduction

Sigma receptors were initially described as novel opioid receptors (Martin et al., 1976) but were later found to be a distinct class of receptors consisting of two subtypes: sigma-1 and sigma-2. The sigma-1 receptor has been identified and cloned for some time (Hanner et al., 1996; Kekuda et al., 1996; Mei and Pasternak, 2001; Abate et al., 2010), with the crystal structure of the trimer being recently reported (Schmidt et al., 2016). The molecular identity of the sigma-2 binding site has only very recently been determined as TMEM97, an endoplasmic reticulum-resident transmembrane protein that regulates the sterol transporter NPC1 (Alon et al., 2017). It has been reported that both subtypes of the sigma receptor, but in particular sigma-2, are overexpressed in rapidly dividing normal cells and in tumour cell lines derived from various tissues (Vilner et al., 1995) highlighting a role in cell growth and proliferation with a potential link to cancer.

Sigma-1 receptors have been well-studied and several functions have been described including: modulation and synthesis of dopamine and acetylcholine (Booth and Baldessarini, 1991; Patrick et al., 1993); modulation of N-methyl-D-aspartate (NMDA)-stimulated neurotransmitter release (Gonzalez-Alvear and Werling, 1995; Monnet et al., 1996); modulation of opioid analgesia (King et al., 1997); and neuroprotective and anti-amnesic activity (Maurice and Lockhart, 1997; Brimson et al., 2018). Sigma-1 receptor antagonists show promise in the treatment (Spruce et al., 2004) and diagnosis (van Waarde et al., 2015) of several cancers. Sigma-2 receptors are mainly involved in the regulation of cell proliferation and viability, with agonists driving changes in cell morphology and a reduction in cell division, leading ultimately to apoptosis (Bowen, 2000).

The current focus on the sigma-2 receptor is underpinned by the observation that its presence not only correlates with the proliferation of tumours but also that it plays an important role in cell survival. *In vitro* studies have shown that sigma-2 ligands can induce apoptosis and hence inhibit tumour growth. As such, it has been proposed that the sigma-2 receptor could be used as both a diagnostic and therapeutic target (van Waarde et al., 2015). Indeed, trials are underway in these areas to determine the potential of the sigma-2 receptor and its ligands in oncology. For example, early trials using radiolabelled sigma-2 ligands in PET imaging have shown success in imaging certain tumours. Furthermore, *in vitro* studies using pancreatic and ovarian cancer cell lines have shown significant increases in the pharmacological effects of chemotherapeutics when used in combination with sigma-2 ligands. Sigma-2 ligands conjugated with anti-cancer drugs are also under development to ensure targeted drug delivery in order to minimise the toxicities associated with chemotherapy (Zeng et al., 2017).

Sigma-1 receptors are usually quantified by radioligand binding assays using the selective ligand (+) pentazocine that binds to the sigma-1 receptor with relatively high affinity. Binding of (+) pentazocine to other proteins appears poor, leading to rapid dissociation from low-affinity sites and little contribution of background or non-specific binding to overall binding. Although described as an agonist, (+) pentazocine binds with a Hill slope of unity which is not affected by the inclusion of GTP or suramin. In contrast, antagonists bind with low Hill slopes. The addition of GTP or suramin causes loss of the high-affinity state of the sigma-1 receptor for the antagonist and leads to a Hill slope of unity being achieved (Brimson et al., 2011).

Problems arise, however, when radioligand binding assays are performed to study the sigma-2 receptor. In this article, we show that this can overestimate the number of sigma-2 receptors present in a system where sigma-1 receptors are also present. This may also explain why sigma-2 receptors are described as ubiquitous (Stracina and Novakova, 2018).

The standard protocol used for identifying and quantifying the sigma-2 receptor relies on the radioligand [^3^H] di-*O*-tolyl guanidine (DTG). DTG is a pan-sigma ligand, binding both receptors with equal affinity. As most binding assays have been performed in tissues or cell lines containing sigma-1 receptors, it has become standard to determine sigma-2 binding in the presence of either (+) pentazocine or dextrallorphan to mask sigma-1 binding sites (Vilner et al., 1995; Chu et al., 2015; Chu and Ruoho, 2015). However, this protocol, whilst fully integrated into the sigma receptor researcher's toolkit, is seriously flawed. Here, we explain the reasons and consequences of relying on a masking protocol and offer alternatives.

## Materials and Methods

### General Materials

Tissue culture media, antibiotics, trypsin, and serum were purchased from Invitrogen (Paisley, UK) or Sigma-Aldrich (Ireland). [^3^H] (+) pentazocine ((1*S*,9*S*,13*S*)-1,13-dimethyl-10-(3-methylbut-2-en-1-yl)-10-azatricyclotrideca-2,4,6-trien-4-ol) and [5-^3^H(N)]- 1,3-di-*O*-tolylguanidine (DTG) were purchased from PerkinElmer (Beaconsfield, UK). Other reagents were purchased from Sigma-Aldrich (Poole, UK). Before use, drugs were dissolved in an appropriate vehicle and diluted in assay buffer. The pH of each solution was adjusted to 7.4.

### Tissue Culture and Membrane Preparation

MDA-MB-468 (ATCC HTB-132) and MCF7 (ATCC HTB-22) breast cancer cell lines were obtained from LGC Promotech, UK. MDA-MB-468 cells were maintained in DMEM, high glucose (41965-062) supplemented with 10% foetal calf serum. MCF7 cells were maintained in MEM (M2279) supplemented with 2 mM L-glutamine and 10% foetal calf serum. Cells were cultured at 37°C in a humidified incubator with 5% CO_2_. To prepare membranes for binding studies, cells were suspended in sigma binding buffer [SBB: 50 mM Tris-HCl; pH 8.0, (Vilner et al., 1995)], sonicated (1 min), and then centrifuged (22,000*g*, 20 min, 4°C). The supernatant was discarded and the pellet suspended in SBB.

### Saturation Binding

#### Sigma-1 Receptor Binding

Assays (total volume 100 µl) were performed using 0–300 nM [^3^H] (+) pentazocine at room temperature for 2 h in SBB as previously described (Vilner et al., 1995). Non-specific binding was determined using 1 mM reduced haloperidol (Schetz et al., 2007). The concentration of reduced haloperidol to determine non-specific binding was higher than in previous studies, as assays described below used higher concentrations of radioligand than used in standard radioligand binding assays. Assays were terminated by addition of ice-cold tris-buffered saline (TBS: 154 mM NaCl, 10 mM Tris, pH 7.4) and filtration through glass fibre filters (GF/B, Sigma-Aldrich, Poole, UK) using a cell harvester. Tubes and filter discs were washed (2 x 3 ml) with ice-cold TBS, and the filter discs dried under vacuum. Scintillation counting was carried out in ProSafe FC+ cocktail (Meridian Biotechnologies Ltd, Tadworth, UK) after overnight incubation.

#### Sigma-2 Receptor Binding

Assays (total volume 100 μl) were performed at room temperature for 4 h with 1–300 nM [^3^H] DTG in SBB. Non-specific binding was determined using 1 mM reduced haloperidol. To investigate the effects of (+) pentazocine, [^3^H] DTG saturation curves were performed in the absence or presence of (+) pentazocine [100 nM (Chu and Ruoho, 2015) or 1 µM (Shiba et al., 2005; Xu et al., 2005)]. Assays were terminated by addition of ice-cold TBS and filtration through glass fibre filters (GF/C, Sigma-Aldrich, Poole, UK) using a cell harvester. Tubes and filter discs were washed with ice-cold TBS (2 x 3 ml) and the filter discs dried under vacuum. Scintillation counting was carried out in ProSafe FC+ cocktail after overnight incubation.

### Competition Binding Assays

Competition binding assays (total volume 100 μl) were performed using a final assay concentration of 50 nM, 100 nM, or 1 µM [^3^H] (+) pentazocine with increasing concentrations of DTG (10 nM–1 mM). Alternatively, 10–30 nM [³H] DTG was employed in the presence of increasing concentrations of (+) pentazocine (10 nM–1 mM). The assay was then allowed to equilibrate at room temperature for 4 h. After equilibration, the membranes were harvested by rapid filtration through GF/B ([^3^H] (+) pentazocine) or GF/C ([^3^H] DTG) glass fibre filters. Tubes and filter discs were washed with ice-cold TBS (2 x 3 ml), and the filter discs dried under vacuum. Non-specific binding was determined using 1 mM reduced haloperidol. Under these conditions less than 10% of either the [^3^H] (+) pentazocine or [^3^H] DTG was bound.

All data were calculated and presented using GraphPad Prism v7.02.

### Modelling of Dextrallorphan Binding

Whilst (+) pentazocine is the masking drug used by most researchers, several publications have used dextrallorphan as an alternative. We were unable to obtain dextrallorphan for these studies and have therefore modelled binding experiments using published data. Methods for and results from the modelling can be found in **Supplementary Material**.

## Results

**Figure 1:**
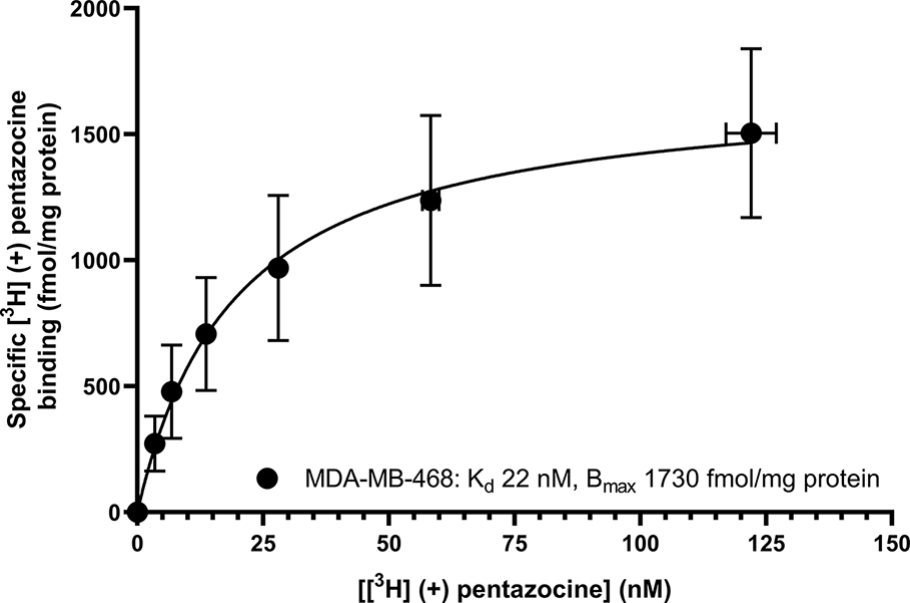
Saturation binding curve for [³H] (+) pentazocine to membranes prepared from MDA-MB-468 cells. Non-specific binding was determined in the presence of 1 mM reduced haloperidol. Data represent mean ± SEM for both binding and radioligand concentration from three independent experiments. No specific binding was observed using [³H] (+) pentazocine and membranes prepared from MCF7 cells.

**Figure 2:**
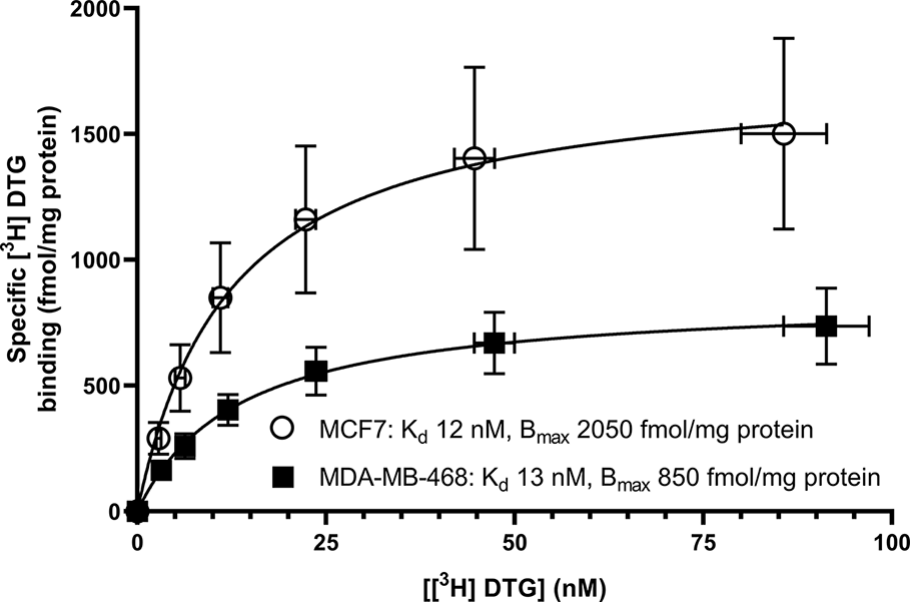
Saturation binding curve for the pan-sigma ligand, [³H] DTG, to membranes prepared from MDA-MB-468 cells (filled squares) and MCF7 cells (open circles). Non-specific binding was determined in the presence of 1 mM reduced haloperidol. Data represent mean ± SEM for both binding and radioligand concentration from three independent experiments.

Saturation binding of [^3^H] (+) pentazocine to membranes prepared from MCF7 and MDA-MB-468 cells was performed. MDA-MB-468 cell membranes showed binding with K_d_ 22 nM (pK_d_ = 7.65 ± 0.13) and B_max_ of 1,730 ± 330 fmol/mg protein (**Figure 1**). There was no specific binding of [³H] (+) pentazocine to MCF7 cell membranes detected (data not shown). Saturation binding curves were also performed using the pan-sigma ligand [³H] DTG, which bound to MCF7 cells with K_d_ 12 nM (pK_d_ = 7.92 ± 0.03, n = 3) and MDA-MD-468 cell membranes with K_d_ 13 nM (pK_d_ 7.88 ± 0.01, n = 3). The B_max_ values were 2,050 ± 100 and 850 ± 200 fmol/mg protein for MCF7 and MDA-MB-468 cells respectively (**Figure 2**).

**Figure 3:**
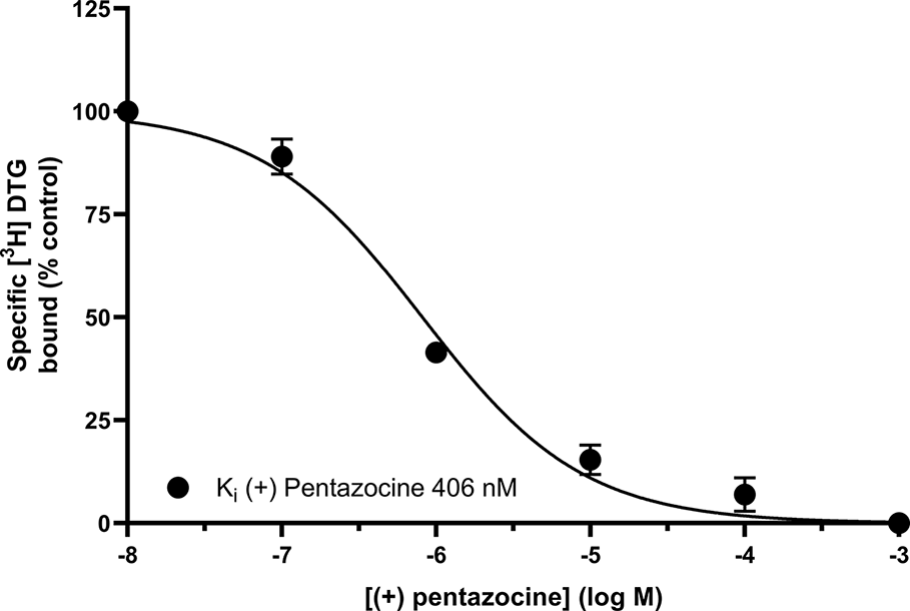
Displacement of [³H] DTG from sigma-2 receptors by (+) pentazocine. Although no binding was detected using [³H] (+) pentazocine, the competition binding curve shows displacement of [³H] DTG from sigma-2 receptors in membranes prepared from MCF7 cells by increasing (+) pentazocine concentrations. Non-specific binding was determined in the presence of 1 mM reduced haloperidol. Data represent mean ± SEM from four independent experiments.

**Figure 3** shows that (+) pentazocine readily competed with the pan-sigma ligand [^3^H] DTG for the sigma-2 receptor. Our assays were performed using membranes prepared from MCF7 cells, which show no specific binding of [^3^H] (+) pentazocine in radioligand binding assays [**Figure 1** and (Vilner et al., 1995)], and so express very few, if any, sigma-1 receptors. Competition assays were performed using low concentrations (10–30 nM) of [³H] DTG. An IC_50_ of 620 nM was determined, resulting in a K_i_ of 406 nM (pK_i_ = 6.39 ± 0.07, n = 4) calculated using the Cheng-Prusoff correction. These results show that whilst the interaction between sigma-2 receptors and (+) pentazocine cannot be shown directly using [³H] (+) pentazocine, there is a clear, measurable interaction.

**Figure 4:**
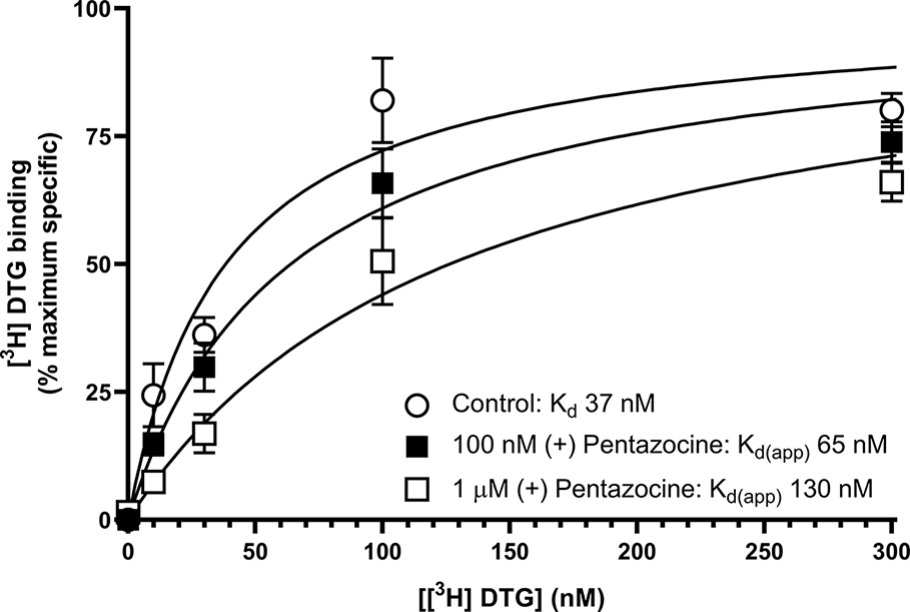
Effect of (+) pentazocine on the saturation binding of [³H] DTG to sigma-2 receptors in MCF7 cell membranes. Saturation binding curves for [³H] DTG to membranes prepared from MCF7 cells were performed in the absence (open circles) or presence of (+) pentazocine (100 nM, filled squares; 1 µM, open squares). Non-specific binding was determined in the presence of 1 mM reduced haloperidol. Data represent mean ± SEM from 11 independent experiments. K_d(app)_ = apparent K_d_ in the presence of competitor.

We next sought to determine whether the inclusion of (+) pentazocine would affect the saturation curve of [³H] DTG, performing the assay in accordance with frequently used protocols (Chu and Ruoho, 2015). Assays were performed using [³H] DTG (1–300 nM) in the absence and presence of (+) pentazocine (100 nM or 1 µM) with membranes prepared from MCF7 cells, which, as highlighted above, show no specific binding of [³H] (+) pentazocine at the concentrations used in radioligand binding assays. A rectangular hyperbolic curve was obtained in all three conditions (**Figure 4**). Using GraphPad Prism to plot the saturation curves allowed comparisons of K_d_, apparent K_d_ and B_max_ values. We have deliberately not presented results in the form of Scatchard plots, as such linear transformations are not considered suitable for statistical analysis or determination of K_d_ or B_max_ values (Rodbard et al., 1980; GraphPad, 2020). These experiments were performed using a different batch of [³H] DTG to that used in the saturation binding experiments described above, and a modest difference in K_d_ for DTG was observed between this experiment (37 nM, pK_d_ = 7.43 ± 0.10, mean ± SEM, n = 11) and the saturation analysis shown in **Figure 2** (12 nM). The highest concentration of DTG used (300 nM) bound 88% of the available receptors based on the rectangular hyperbolic fit observed in **Figure 2**. As expected, the inclusion of 100 nM (+) pentazocine did not affect B_max_ calculations. The apparent K_d_ was moderately increased to 65 nM (pK_d_ = 7.19 ± 0.09, mean ± SEM, n = 11) with DTG (300 nM) binding 82% of the available receptors. Inclusion of the higher concentration of (+) pentazocine (1 µM) again did not affect the calculated B_max_. However, the apparent K_d_ was shifted even higher: 130 nM (pK_d_ = 6.89 ± 0.09, mean ± SEM, n = 11) and DTG (300 nM) binding 71% of the receptors available. These data show that the frequently used protocol for establishing K_d_ and B_max_ for sigma-2 receptors would give a raised K_d_ value for DTG, whilst recognising all sigma-2 receptors in the system.

**Figure 5:**
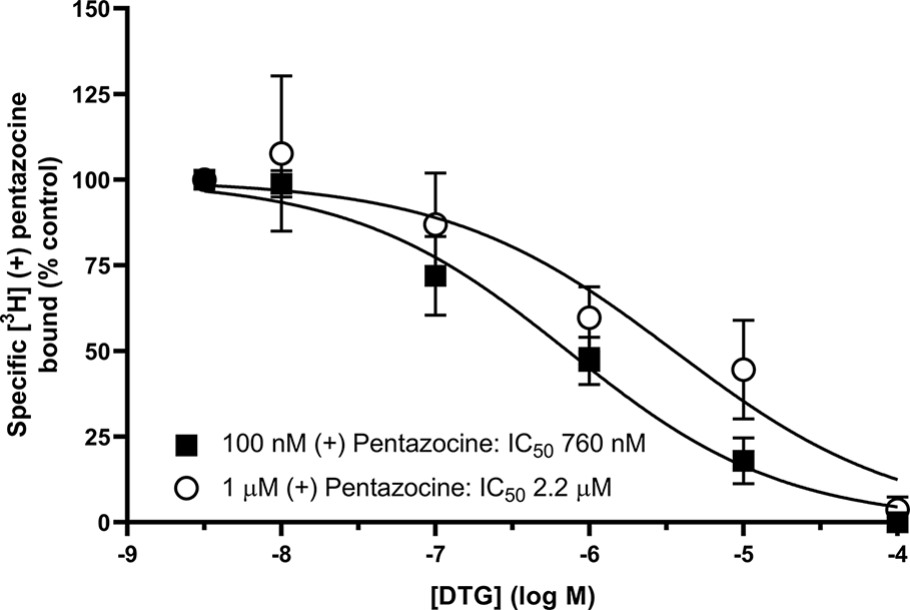
Displacement of [³H] (+) pentazocine from sigma-1 receptors by DTG. Competition binding curve showing the displacement of either 100 nM (filled squares) or 1 µM (open circles) [³H] (+) pentazocine from sigma-1 receptors in membranes prepared from MDA-MB-468 cells by increasing DTG concentrations. Non-specific binding was determined in the presence of 1 mM reduced haloperidol. Data represent mean ± SEM from 8 (100 nM [³H] (+) pentazocine) or 6 (1 µM [³H] (+) pentazocine) independent experiments.

We also investigated whether [³H] DTG could compete with the *masking agent* (+) pentazocine and bind to sigma-1 receptors. In order to observe loss of (+) pentazocine binding to these sites, experiments were performed in membranes prepared from MDA-MB-468 cells. Incubations of [³H] (+) pentazocine (100 nM and 1 µM) were performed with the inclusion of increasing concentrations of DTG. Radioligand binding assays are rarely performed with the concentrations of radioligand used here. Preparations of [³H] (+) pentazocine were mixed with unlabelled (+) pentazocine to obtain stocks suitable for these binding studies. The binding of 100 nM [³H] (+) pentazocine was reduced by increasing concentrations of DTG with an IC_50_ of 760 nM (pIC_50_ = 6.1 ± 0.2, mean ± SEM, n = 8). When considering data using 1 µM [³H] (+) pentazocine, DTG was, as expected, less effective, with an IC_50_ of 2.2 µM (pIC_50_ = 5.7 ± 0.4, mean ± SEM, n = 6) (**Figure 5**). Using the Cheng-Prusoff correction, K_i_ values of 137 and 47 nM for DTG can be calculated at 100 nM and 1 µM [³H] (+) pentazocine respectively. Interpolation of the curves allows calculation of the amount of [³H] (+) pentazocine displaced from the sigma-1 sites at different concentrations of DTG (**Table 1**). Data show that 37% of 100 nM [³H] (+) pentazocine and 17% of 1 µM [³H] (+) pentazocine was displaced by 300 nM DTG. These data show that under conditions frequently used, up to 37% of sigma-1 receptors present would contribute to the DTG signal and inflate the B_max_ value. In extreme circumstances, this could account for all the binding observed.

**Table 1:** Displacement of [³H] (+) pentazocine from sigma-1 receptors by increasing concentrations of DTG.

[[³H] (+) pentazocine]	[DTG] 3 nM	[DTG] 10 nM	[DTG] 30 nM	[DTG] 100 nM	[DTG] 300 nM
100 nM	2.5 ± 0.9	4.7 ± 1.6	8.6 ± 2.6	26.3 ± 9.9	37 ± 11
1 µM	2.9 ± 2.5	4.8 ± 3.9	7.6 ± 5.9	11.6 ± 8.2	17 ± 11

Data represent [³H] (+) pentazocine displaced by increasing concentrations of DTG (as % of binding in the absence of DTG). Data (mean ± SEM) are interpolated from individual competition curves, n = 8 (100 nM (+) pentazocine) and n = 6 (1 µM (+) pentazocine). Values are derived using the data presented in **Figure 5**.

**Figure 6:**
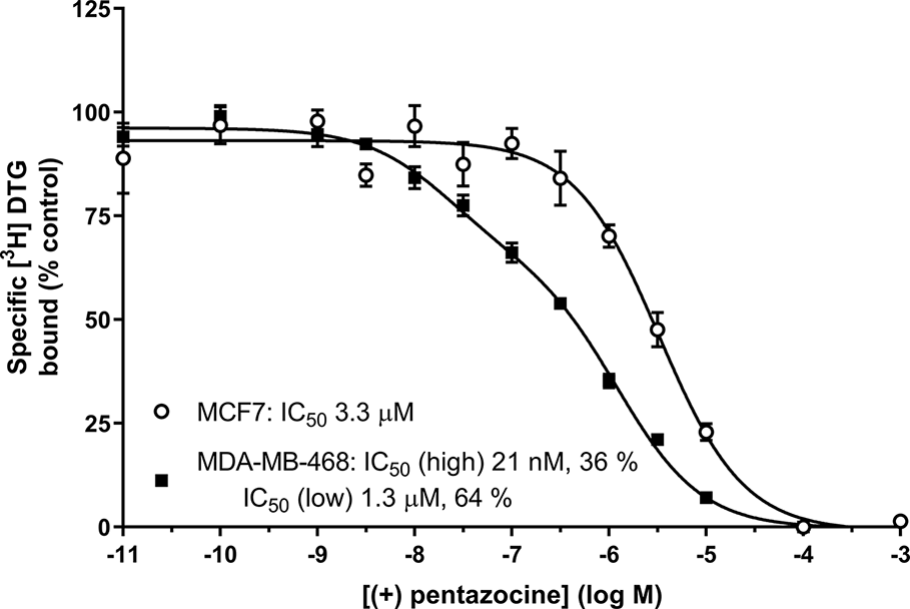
Displacement of [³H] DTG from sigma receptors by (+) pentazocine. Competition binding curve showing the displacement of [³H] DTG from membranes prepared from MDA-MB-468 cells (filled squares) and MCF7 cells (open circles) by increasing (+) pentazocine concentrations. Non-specific binding was determined in the presence of 1 mM reduced haloperidol. Data represent mean ± SEM from 5 (MDA-MB-468) and 4 (MCF7) independent experiments. Curve fitting was achieved comparing a one- and two-site fit (GraphPad Prism).

We offer one possible remedy to the current problem of assessing levels of sigma-1 and sigma-2 receptors in cells and tissues in the absence of commercially available sigma-2 receptor-selective radioligands: **Figure 6** shows competition binding between a fixed concentration of [³H] DTG and a range of concentrations of unlabelled (+) pentazocine in MDA-MB-468 and MCF7 cell membranes. A monophasic competition curve is observed in MCF7 cell membranes. This indicates a single, low affinity site (IC_50_ 3.3 µM) is present. In contrast, MDA-MB-468 cells show the existence of sites with high- and low-affinity for (+) pentazocine. Two-site analysis (GraphPad Prism) identifies that 36% of these sites had high affinity (IC_50_ 21 nM), indicating these are sigma-1 receptors, with the remaining 64% with a low affinity (IC_50_ 1.3 µM), representing sigma-2 receptors.

We note that not all groups use (+) pentazocine to mask sigma-1 receptors. Indeed, several publications have used dextrallorphan. Unfortunately, we have not been able to obtain this ligand but have used modelling to determine whether this may provide a better sigma-1 receptor mask than (+) pentazocine. Results presented in **Supplementary Material** suggest that 1 µM dextrallorphan would bind 2.2–6.3% of the sigma-2 receptors. The addition of 300 nM DTG would displace 88% of this binding. However, DTG would also displace dextrallorphan from the sigma-1 receptor, rendering only 71.5–86.1% of sigma-1 receptors masked (see **Supplementary Material**).

From the above data, it is clear that none of the masking protocols widely accepted should be used. Addition of DTG will compete with the binding of (+) pentazocine and dextrallorphan to both sigma-1 and sigma-2 sites.

## Discussion and Conclusions

The results presented here are in keeping with previously published data on all of the agents used. Our affinity of 22 nM for (+) pentazocine at the sigma-1 receptor is both internally and externally consistent. We have previously shown affinities of 7.7 nM (Spruce et al., 2004) and 17 nM (Brimson et al., 2011) (obtained from MDA-MB-468 membranes and permeabilised cells, respectively). A selection of data from other studies with a variety of tissues and conditions gives overlapping results: guinea pig liver microsomes (0.8 nM) (Hanner et al., 1996); mouse lung membranes (1.4 nM) (Lever et al., 2015); guinea pig brain membranes (1.6 nM obtained by means of homologous competition) (Xu et al., 2015); mouse brain homogenates (5.1 nM) (Langa et al., 2003); bovine adrenal medullar membranes (18 nM) (Paul et al., 1993); and rat cerebral membranes (19.9 nM) (Shiba et al., 2005). It is recognised that differences in the sigma-1 receptor sequences from different species may contribute to some variation in affinity. Only one of the above studies (Hanner et al., 1996) used recombinant protein with a known sequence. In the present study, [³H] DTG gave a B_max_ of 2,050 ± 100 fmol/mg protein of sigma receptors in MCF7 cells. In the absence of any measureable specific [³H] (+) pentazocine binding, we consider these to be sigma-2 receptors. This is in agreement with previously published data of 2,071 fmol/mg protein (Vilner et al., 1995) in these cells. It is, of course, possible that low levels of sigma-1 receptors are expressed in these MCF7 cells obtained from ATCC. Indeed, Western blotting and immunocytochemical analysis of MCF7 cells obtained from ECACC (Radif et al., 2018) demonstrated the presence of sigma-1 receptors but to our knowledge, no direct comparison of MCF7 cells from these different sources has been made.

The inclusion of (+) pentazocine in binding assays using MCF7 cells caused a rightward shift in the K_d_ of [³H] DTG binding, but had no effect on B_max_ calculations. This is unsurprising as (+) pentazocine and [³H] DTG are competing at the sigma-2 receptors. However, the percentage of sigma-2 receptors bound by DTG at 300 nM DTG falls from 88% (no (+) pentazocine) to 71% in the presence of 1 µM (+) pentazocine, based on interpolation of values shown in **Figure 4**. Our K_d_ values of DTG for the sigma-2 receptor, 12-37 nM, are difficult to compare with previous data, as most reported data have been made in the presence of 100 nM–1 µM (+) pentazocine or 1 µM dextrallorphan. Examples include: 22.3 nM (Shiba et al., 2005); 25 nM (Vilner et al., 1995); 30.7 nM (Xu et al., 2005); 39.9 nM (Lever et al., 2006); and 74.8–91.1 nM (Chu et al., 2015). In this respect, our data are in keeping with, and highly comparable to, previous data. As such, our conclusions should be considered relevant to all using these reagents.

The affinity of (+) pentazocine for the sigma-2 receptor has been reported in a number of published studies. However, data in the present study, along with consideration of the methods used in many of the previous studies suggest that a re-evaluation might be appropriate. For example: a K_d_ of 56 nM was reported for (+) pentazocine at the sigma-2 receptor in guinea pig brain homogenates (‘in the presence of an excess of non-radiolabelled (+) pentazocine for selective masking of sigma-1 receptors') (Sunnam et al., 2011); 327 nM in rat liver homogenate (in the presence of 100 nM (+) pentazocine to mask sigma-1 receptors) (Zampieri et al., 2009); 728 nM in guinea pig brain membranes (in the presence of 200 nM (+) pentazocine) (Lever et al., 2006); 1,440 nM in rat brain homogenates (in the presence of 1 µM (+) pentazocine) (Xu et al., 2005); and 2680 nM in rat cerebral membranes (in the presence of 1 µM (+) pentazocine) (Shiba et al., 2005). Values reported in the absence of a masking concentration of (+) pentazocine include: 1.7–3.3 µM (reported in C6 and NG115-08 cells in the absence of masking agent; these cells were reported as having a very low density of high-affinity (+) pentazocine binding sites) and 2.1–9.4 µM (in the presence of 1 µM dextrallorphan) (Vilner et al., 1995). Thus, although the affinity of (+) pentazocine has been widely reported, the effects of this relatively low-affinity binding have been underestimated.

Our experimental data show the apparent K_d_ of [³H] DTG binding to the sigma-2 receptor increases with increasing (+) pentazocine concentration. Equally, the calculation of K_i_ values for novel compounds acting at sigma-2 receptors would be complicated, as DTG, (+) pentazocine and the test compound will be competing at the same site. There may be additional complications if compounds also bind to the sigma-1 receptor with high affinity. If this results in substantial ligand depletion, estimates of affinity at the sigma-2 receptor would be compromised. We would suggest that ideally, determination of binding parameters for the sigma-2 receptor would be best performed using [³H] DTG and cell preparations devoid of sigma-1 receptors, thereby avoiding the need for a masking agent. This could include, for example, MCF7 cell membranes, although caution should be applied as we have found some of these to contain sigma-1 receptors (Spruce et al., 2004; Radif et al., 2018).

The consequences of the low affinity binding of (+) pentazocine to sigma-2 receptors are likely to be minimal when reporting B_max_ values for these receptors in membrane preparations. However, the ability of DTG to compete with (+) pentazocine for sigma-1 receptors is a major concern when (+) pentazocine is being used as a mask for sigma-1 receptors. To date, we are unaware of any cell or tissue reported as lacking sigma-2 receptors but we suggest that determination of sigma-2 sites should be revisited. Data presented here bring into question the previously accepted method of calculating sigma-2 receptor levels in cell lines using [³H] DTG in the presence of either (+) pentazocine or dextrallorphan to mask sigma-1 receptors. Indeed, our data clearly highlight that the number of sigma-2 receptors will have been overestimated using such methodology, as it is likely that, if present, sigma-1 receptors will also have bound [³H] DTG.

In terms of any underlying biology, there is little rationale for comparisons between sigma-1 and sigma-2 receptors as, after all, they are very different proteins with very different roles. Despite this, a reliable protocol for determination of sigma-2 sites in the presence of sigma-1 receptors is required. Thus, we propose that alternative methodology is employed to quantitate sigma-2 receptor levels, specifically where masking agents are excluded. The use of (+) pentazocine for the quantitation of sigma-1 receptors is greatly entrenched in our research methodologies. Unless a direct comparison of sigma-1 and sigma-2 receptor levels is required, an ideal way would be to use sigma-2 receptor selective tools (Zeng et al., 2017). However, until these agents are commercially available, we suggest that an alternative would be to use [^3^H] DTG to obtain levels of total sigma binding sites (i.e., sigma-1 plus sigma-2 receptors) accompanied by competition binding with (+) pentazocine or another agent selective for one particular receptor. Subsequent two-site analyses would then allow determination of the relative amounts of each target. Such methodology has been used previously: in a rarely cited paper (Kovacs and Larson, 1995) it was shown that [³H] DTG can be used to label all sigma sites. Using sigma-1-selective agents a biphasic competition curve was demonstrated, equivalent to sigma-1 and sigma-2 sites. Computer assisted data analysis (e.g. GraphPad Prism, as used here) is sufficiently developed to determine the presence of even a relatively small proportion of a second (affinity) binding site.

Although the methodology described above may provide an alternative strategy for determination of sigma-1 and sigma-2 receptors, this approach has generated inconsistencies. Kovacs and Larson (1995) showed that in spinal cord, [³H] DTG alone bound 150% of the sites labelled by [³H] (+) pentazocine, suggesting there were twice as many sigma-1 receptors as sigma-2 receptors. However, when the competition experiments were performed, these suggested the reverse, as (+) pentazocine only displaced 30% of the [³H] DTG with high affinity (Kovacs and Larson, 1995). Similarly, [³H] DTG bound to fewer sites than [³H] (+) pentazocine in several regions of the brain (Walker et al., 1990). Our results with MDA-MB-468 cells also show this discrepancy: **Figure 1** shows that MDA-MB-468 cells had a B_max_ of 1730 fmol/mg protein for [^3^H] (+) pentazocine and only 850 fmol/mg protein for the pan-sigma ligand [³H] DTG. **Figure 5** then shows that 36% of these [³H] DTG binding sites were sigma-1 receptors. Whether such discrepancies arise as a consequence of, for example, the given values of radioligand specific activity, breakdown of the radioligand, tritium exchange between the ligand, and other constituents (including water) or the presence of labelled precursor molecules (Lazareno and Birdsall, 2000) remains to be established. Until that point, this protocol would benefit from further consideration before it is widely accepted.

In addition to the issues described above, two recent publications highlight the possibility that [^3^H] DTG binds to something other than sigma-2 receptors. Thus, knock-out of the recently identified sigma-2 binding site, TMEM97, in HeLa cells showed residual binding sites for [³H] DTG (Riad et al., 2018; Zeng et al., 2019). These sites had an apparent K_d_ for DTG of 300–400 nM, with assays performed in the presence of 1 μM (+) pentazocine (to *mask* the sigma-1 receptor). Utilising a derivation of the Michaelis-Menten equation for a competitive antagonist (K_d(app)_ = K_d_*(1 + [I]/K_i_), where K_d(app)_ is the apparent K_d_ of the radioligand in the presence of a competitor at fixed concentration) and the values given in this paper (DTG K_d_ = 12 nM, (+) pentazocine K_i_ (sigma-1 receptor) = 22 nM, [(+) pentazocine] = 1 μM), the calculated K_d(app)_ for DTG is 557 nM, which is well within experimental error for suggesting that this residual binding site may be the sigma-1 receptor, despite the suggestion that this is unlikely (Riad et al., 2018). It is noteworthy that RHM-4, a selective sigma-2 receptor ligand [K_i_ 8.2 nM and 12,900 nM for sigma-2 and sigma-1 receptors, respectively (Hou et al., 2006; Zeng et al., 2019)] was unable to detect this residual binding when used as the radioligand. In this way, [^125^I] RHM-4 binding the sigma-2 receptor reflects the beauty of [^3^H] (+) pentazocine when studying the sigma-1 receptor. [^125^I] RHM-4 binds the sigma-2 receptor with sufficient dwell time to monitor the interaction readily. Rapid dissociation from the alternative binding sites with low affinity (in this case, sigma-1 receptors) means they do not contribute to any directly observable radioactive signal.

The more recent molecular identification of the sigma-2 receptor (Alon et al., 2017) has allowed the generation of cell lines lacking either sigma-1 receptors (Mavlyutov et al., 2017) or sigma-2 receptors (Riad et al., 2018). Such cell lines may well prove to be useful in dissecting out the cellular roles of the individual receptor types and contribute to the development of more selective ligands for pharmacological and therapeutic use. Furthermore, despite limitations of the models, the availability of knockout mice, such as that for the sigma 1 receptor (Langa et al., 2003), will undoubtedly contribute to a full understanding of the pathophysiological roles of these receptors. Such developments will certainly add to the available tools and methodologies. However, the present importance of identifying and quantifying sigma receptors (particularly sigma-2 receptors), potentially for tumour imaging and as a molecular target in cancer (see Introduction), highlight that robust methodology must be in place. We hope that the work presented here will sound a note of caution with current methodologies and highlight the need for further consideration and development.

## Author’s Note

**Figures 1** and **6**, and **Supplementary Figures 1, 2, 3** and **4** appear in preliminary form in the PhD thesis by HA (Expression of sigma receptors in human cancer cell lines and effects of novel sigma-2 ligands on their proliferation, University of Wolverhampton, 2018).

## Data Availability Statement

The datasets generated for this study are available on request to the corresponding author.

## Author Contributions

HA, PB, GW and SS were involved in performing the experiments described. HA, DF and SS contributed to the modelling. All authors were involved in drafting and preparation of this manuscript.

## Funding

We would like to thank Dudley Group of Hospitals NHS Trust, New Cross Hospital NHS Trust and RCSI-Bahrain for the financial support.

## Conflict of Interest

Author DF is employed by Eli Lilly.

The remaining authors declare that the research was conducted in the absence of any commercial or financial relationships that could be construed as a potential conflict of interest.
