# Supplementary material for: Hazards of Using Masking Protocols When Performing Ligand Binding Assays: Lessons From the Sigma-1 and Sigma-2 Receptors
Source: Front Pharmacol. 2020 Mar 13;11:309. doi: 10.3389/fphar.2020.00309 (PMC7082753; doi:10.3389/fphar.2020.00309)
Supplement: Supplementary file 1 [file DataSheet_1.docx]

Supplementary Material

**Supplementary Methods.**

**Modelling of dextrallorphan binding**

Whilst (+) pentazocine is the masking drug used by most researchers, several publications have used dextrallorphan as an alternative. Here, we model the effects of dextrallorphan to determine whether it is an appropriate substitute for (+) pentazocine. We have used two affinities of dextrallorphan previously reported for the sigma-1 receptor: K_i_ of 16.1 nM (Hellewell and Bowen, 1990); alternatively, an IC_50_ of 163 nM has been reported for dextrallorphan against 1-2 nM of [³H] (+)-3-(3-hydroxyphenyl)-N-(1-propyl)piperidine (PPP), which was reported to have a K_d_ of 30 nM (Largent et al., 1987). Using the Cheng-Prusoff correction, this enables us to calculate a K_i_ for dextrallorphan of 153-158 nM. The affinity of dextrallorphan for the sigma-2 receptor has not been defined and we have estimated this from the only data that we have found for this ligand (Hellewell and Bowen, 1990). These data were obtained using [³H] DTG and [³H] PPP in PC12 cells in which 5 μM dextrallorphan is described as displacing less than 30 % of [³H] DTG or [³H] PPP (Hellewell and Bowen, 1990). As a similar statement regarding other drugs showing less than 30 % displacement at 10 μM is made in the same paper, we have estimated the affinity based on 30 % displacement of 5 nM DTG (K_d_ = 23.7 nM) and 3 nM PPP (K_d_ = 86.3 nM) (Hellewell and Bowen, 1990). These experiments were performed using PC12 cells which appeared to only have sigma-2 receptors, as both DTG and PPP binding was displaced by (+) pentazocine with low-affinity (Hellewell and Bowen, 1990). Entering these values into Equation 1 gives K_30_ (as only 30 % was displaced) values for dextrallorphan of between 6550 nM (calculated using PPP) and 18760 nM (calculated using DTG). From a rectangular hyperbolic binding curve (Equation 2), 30 % binding corresponds to a concentration 42 % of the K_i_. Thus, we estimate the K_i_ of dextrallorphan for the sigma-2 receptor at between 15 μM and 44.7 μM. These estimates for the affinity of dextrallorphan gives a “worst case” scenario; it is accepted that the affinity could be lower. We have used Equation 2 to model saturation binding data using these two estimates of dextrallorphan affinity (16.1 nM and 155 nM) in order to calculate saturation binding to the sigma-1 receptor. Similarly, we have modelled dextrallorphan binding to the sigma-2 receptor using the affinity estimates of 15 μM and 44.7 μM. We have also modelled the ability of [³H] DTG to compete with dextrallorphan at both sites, fitting the data to Equation 3. For our comparisons, we have used a single source for the affinity of DTG binding to either the sigma-1 receptor (K_i_ 35.5 nM) or sigma-2 receptor (K_i_ 39.9 nM); these values were obtained using English Hartley guinea pig brain membranes (Lever et al., 2006).

**Calculation of proportion bound (Equation 1)**

Using a derivation of the Cheng-Prusoff correction (Cheng and Prusoff, 1973), the proportion of inhibition of binding of a ligand can be determined based on the affinities and concentration of each ligand using the equation f_i_ = [I]/([I] + K_i_(1 + LT/K_d_)) (Attie and Raines, 1995), where f_i_ = fraction inhibition, [I] = concentration of added unlabelled drug, K_i_ = affinity of inhibitor, LT = total labelled drug added (assuming no depletion), K_d_ = affinity of labelled drug (equilibrium dissociation constant).

**Saturation binding curves (Equation 2)**

Calculations for saturation binding are based on non-linear analysis of idealised data. This has been performed for ligands with a known Hill slope of unity, using the equation Y = B_max_*[X]/(K_d_ + [X]), where Y = amount bound, B_max_ = maximal binding of the ligand, [X] = concentration of drug analysed, K_d_ = affinity of drug analysed.

**Competition binding curves (Equation 3)**

Competition binding data were analysed using the equation logIC_50_ = log(K_i_*(1+[Radioligand]/K_d_)), where IC_50_ = effective concentration of competing drug able to displace 50 % of the radioligand (present at one concentration), K_i_ = affinity of competing drug, K_d_ = affinity of radioligand.

All data were modelled using GraphPad Prism v7.02.

**Results**

As the affinity of dextrallorphan for the sigma-2 receptor has not been defined, we have used values calculated from Hellewell and Bowen (Hellewell and Bowen, 1990) of 15 μM and 44.7 μM (see Supplementary Methods). These values were then used to model the binding of dextrallorphan to sigma-2 receptors and determine how much will bind under particular experimental conditions. Modelling rectangular hyperbolae with these affinities demonstrated that 1 μM dextrallorphan will bind between 6.3 % (taking an affinity of 15 μM) and 2.2 % (taking an affinity of 44.7 μM) of the sigma-2 receptors (Supplementary Figure 1, Supplementary Table 1).

The published affinity of dextrallorphan for the sigma-1 receptor shows a K_i_ of 16.1 nM (Hellewell and Bowen, 1990). From a second paper (Largent et al., 1987), we have also calculated a K_i_ of 153‑158 nM. We have modelled data using these two affinities (16.1 nM and 155 nM) in order to estimate binding achieved when the *masking* protocol is used (Supplementary Figure 2). Modelling the dissociation of dextrallorphan at 1 μM with affinities of either 16.1 nM or 155 nM using a range of DTG concentrations (0-300 nM) with an affinity of 39.9 nM for the sigma-1 receptor (Lever et al., 2006) shows that DTG will compete with dextrallorphan at the sigma-1 receptor with increasing effect. Thus, at each concentration of DTG different amounts of dextrallorphan will be displaced from the sigma-1 receptor (Supplementary Figure 3). As anticipated, DTG readily displaces dextrallorphan from the sigma-2 receptor (Supplementary Figure 4). These data demonstrate that 1 μM dextrallorphan will bind 97.8 % of the sigma-1 receptors (using 16.1 nM affinity). Addition of 300 nM DTG would displace dextrallorphan from 11.9 % of these sigma-1 sites. This would only permit 86.1 % (97.8 – 97.8*11.9/100) of the sigma-1 receptors to be masked by dextrallorphan. If the lower affinity of dextrallorphan is used (155 nM), 86.3 % of the sigma-1 receptors present would be initially bound (Supplementary Figure 4, Supplementary Table 1). Addition of DTG at 300 nM would displace dextrallorphan and bind to 17.2 % of these sigma-1 sites. Under these conditions only 71.5 % (86.3 – 86.3*17.2/100) of the sigma-1 receptors would be masked by dextrallorphan.

**Discussion and conclusion**

At a concentration of 1 µM, there would be little interaction between dextrallorphan and the sigma-2 receptor. Indeed, based on values previously published, we model around 2-6 % of the sites would be bound. The addition of 300 nM DTG would readily reduce this by around 88 %. The interaction between DTG and the sigma-1 receptor causes a greater problem: as seen with (+) pentazocine, DTG is able to compete with dextrallorphan for sigma-1 receptors. Under the standard protocols presented (1 µM dextrallorphan, up to 300 nM DTG), [³H] DTG will also bind sigma-1 receptors.

**Reference List**

Attie, A.D., and Raines, R.T. (1995). Analysis of Receptor-Ligand Interactions. *J Chem Educ* 72(2)**,** 119-124. doi: 10.1021/ed072p119.

Cheng, Y., and Prusoff, W.H. (1973). Relationship between the inhibition constant (*K*_1_) and the concentration of inhibitor which causes 50 per cent inhibition (*I*_50_) of an enzymatic reaction. *Biochem Pharmacol* 22(23)**,** 3099-3108. doi: 10.1016/0006-2952(73)90196-2

Hellewell, S.B., and Bowen, W.D. (1990). A sigma-like binding site in rat pheochromocytoma (PC12) cells: decreased affinity for (+)-benzomorphans and lower molecular weight suggest a different sigma receptor form from that of guinea pig brain. *Brain Res* 527(2)**,** 244-253. doi: 10.1016/0006-8993(90)91143-5

Largent, B.L., Wikstrom, H., Gundlach, A.L., and Snyder, S.H. (1987). Structural Determinants of Omicron-Receptor Affinity. *Mol Pharmacol* 32(6)**,** 772-784.

Lever, J.R., Gustafson, J.L., Xu, R., Allmon, R.L., and Lever, S.Z. (2006). Sigma1 and sigma2 receptor binding affinity and selectivity of SA4503 and fluoroethyl SA4503. *Synapse* 59(6)**,** 350-358. doi: 10.1002/syn.20253.

**Supplementary Table 1: Modelled dextrallorphan binding to sigma receptors and displacement by DTG.**

These data summarise the calculated percentage occupancy of sigma-1 and sigma-2 receptors by dextrallorphan (1 µM), using affinities either obtained from the literature or calculated here (see Supplementary Methods). In addition, the displacement of that dextrallorphan binding by DTG (300 nM, the concentration widely used in saturation binding assays) is presented.

| **sigma-1 affinity (nM)** | **% sigma-1 binding** | **% displaced by 300 nM DTG** |  | **sigma-2 affinity (nM)** | **% sigma-2 binding** | **% displaced by 300 nM DTG** |
| --- | --- | --- | --- | --- | --- | --- |
| 16.1 | 97.8 | 11.9 |  | 15,000 | 6.3 | 87.6 |
| 155 | 86.3 | 17.2 |  | 44,700 | 2.2 | 88.0 |

Supplementary Figure 1: Calculated binding of dextrallorphan to sigma-2 receptors.

The binding of dextrallorphan to sigma-2 sites was calculated using an affinity estimate of either 15 μM (highest calculated affinity for sigma-2 receptors using data from binding experiments with PPP) and 44.7 μM (highest calculated affinity for sigma-2 receptor using data from binding experiments with DTG) (see Supplementary Methods for further details). The dotted vertical line shows the commonly used concentration of dextrallorphan (1 µM) to mask the sigma-1 receptor.

Supplementary Figure 2: Calculated binding of dextrallorphan to sigma-1 receptors.

The binding of dextrallorphan to sigma-1 receptors was calculated using an affinity estimate of either 16.1 nM (highest reported affinity for sigma-1 receptor) or 155 nM (lowest calculated affinity for sigma-1 receptor) (see Supplementary Methods for further details). The dotted vertical line shows the commonly used concentration of dextrallorphan (1 µM) to mask the sigma-1 receptor.

Supplementary Figure 3: Calculated displacement of dextrallorphan from sigma-1 receptors by DTG.

The displacement of dextrallorphan (1 μM) from sigma-1 receptors by 0-300 nM DTG (concentrations used when performing saturation binding curves for the sigma-2 receptor) was calculated. Affinities of dextrallorphan for sigma-1 receptors, along with the affinity used for DTG are presented in Supplementary Methods.

Supplementary Figure 4: Calculated displacement of dextrallorphan from sigma-2 receptors by DTG.

The displacement of dextrallorphan (1 μM) from sigma-2 receptors by 0-300 nM DTG (concentrations used when performing saturation binding curves for the sigma-2 receptor) was calculated. Affinities of dextrallorphan for sigma-2 receptors, along with the affinity used for DTG are presented in Supplementary Methods.
